# Informing a Canadian paramedic profile: framing concepts, roles and crosscutting themes

**DOI:** 10.1186/s12913-016-1739-1

**Published:** 2016-09-07

**Authors:** Walter Tavares, Ron Bowles, Becky Donelon

**Affiliations:** 1 Centennial College, School of Community and Health Studies, 941 Progress Ave. Station A, P.O. Box 631, M1K 5E9 Toronto, ON Canada; 2 York Region Paramedic Services, 80 Bales Dr. E. R.R.#1, L0G 1V0 East Sharon, ON Canada; 3 Ornge Transport Medicine, 5310, Mississauga, L4W 5H8 ON Canada; 4 Paramedic Association of Canada, 201-4 Florence St., K2P 0W7 Ottawa, ON Canada; 5 McMaster University, 1200 Main St., L8N 3Z5 Hamilton, ON Canada; 6 Justice Institute of British Columbia, 715 McBride Blvd, V3L 5T4 New Westminster, BC Canada; 7 Society for Prehospital Educators of Canada, 101-265 Carleton Dr., T8N 4J9 St. Albert, AB Canada; 8 Alberta College of Paramedics, 2755 Broadmoor Blvd., T8H 2W7 Sherwood Park, AB Canada

**Keywords:** Paramedic, Community paramedic, Health services, Roles framework, Competency profile, Allied health personnel, Primary care, Education, Emergency medical services

## Abstract

**Background:**

Paramedicine is a rapidly evolving health profession with increasing responsibilities and contributions to healthcare. This rapid growth has left the profession with unclear professional and clinical boundaries. Existing defining frameworks may no longer align with the practice of paramedicine or expectations of the public. The purpose of this study was to explore the roles paramedics in Canada are to embody and that align with or support the rapid and ongoing evolution of the profession.

**Methods:**

We used a concurrent mixed methods study design involving a focused discourse analysis (i.e., analysis of language used to describe paramedics and paramedicine) of peer reviewed and grey literature (Phase 1) and in-depth one-on-one semi-structured interviews with key informants in Canadian paramedicine (Phase 2). Data from both methods were analyzed simultaneously throughout and after being merged using inductive thematic analysis.

**Results:**

Saturation was reached after 99 national and international grey and peer reviewed publications and 20 in depth interviews with stakeholders representing six provinces, seven different service/agency types, 11 operational roles and seven provider roles. After merging both data sets three framing concepts, six roles and four crosscutting themes emerged that may be significant to both present-day practice and aspirational. Framing concepts, which provide context, include variable contexts or practice, embedded relationships and a health and social continuum. Roles include clinician, health and social advocate, team member, educator, professional and reflective practitioner. Crosscutting themes including patient safety, adaptability, compassion and communication appear to exist in all roles.

**Conclusions:**

The paramedic profession is experiencing a shift that appears to deviate or at least place a tension on traditional views or models of practice. Underlying and evolving notions of practice are resulting in intended or actual clinical and professional boundaries that may require the profession to re-think how it is defined and/or shaped. Until these framing concepts, roles and crosscutting themes are fully understood, tested and operationalized, tensions between guiding frameworks and actual or intended practice may persist.

## Background

Paramedicine has experienced significant growth in recent years. This health profession is moving beyond a purely emergency or acute care based system, to offering in addition, more complex and integrated health care [1–3]. This shift has largely been driven by growing health care system pressures, and evolving views on how health care is conceptualized and delivered, including for example, de-institutionalizing care and reducing in-hospital time for patients [4, 5]. Increasingly, paramedicine has become recognized as a health profession whose growing clinical acumen and unique point of contact with the public/patients can be leveraged to better serve their health care needs and meet policy goals; not only for those who contact paramedic services in a time of acute or emergent crisis (as is traditionally the case), but also for broader patient groups and needs [1, 6–8]. As a result, evolution of the profession has been rapid, with many trying to catch up by rethinking and reevaluating the boundaries of paramedicine.

As paramedicine becomes part of system advances, well-established practice boundaries become unclear [8]. For instance, in contrast to more “traditional” responsibilities (e.g., responding solely to emergencies), paramedics have played a large part in the delivery of healthcare in under-serviced areas, or been integrated into long-term care settings as well as primary and/or family health teams [9–12]. Alternative disposition for patients (e.g., bypass protocols, referrals, treat and release programs) are increasingly common [13–16]. Even in more traditional models, innovative, specialized and expanding care programs are emerging [14, 17, 18]. These “non-traditional” practice settings have been effective in helping to manage acute and chronic conditions, reduce unplanned transfers to emergency departments, promote more health care touch points for patients and avoid or minimize admission and readmission rates [19]. These advances reflect conceptual and operational shifts in the profession, with significant implications on the abilities required of those who practice within it.

For Canada, the Paramedic Association of Canada’s (PAC) National Occupational Competency Profile (NOCP) defines Canadian paramedic practice nationally by describing the *competencies* or tasks paramedics are expected to possess when practicing at a specified level (i.e., primary, advanced and critical care) [20]. While the NOCPs have been seminal in helping to describe paramedicine in Canada, the emphasis on decontextualized skills, the profession’s rapid growth and the emergence of increasingly diverse clinical programs, has led to a framework that may now be poorly aligned with the actual experiences of most paramedics and/or the public. Further, the NOCP is limited to specification of what paramedics can do, rather than what *roles* (i.e., professional capabilities) paramedics are to embody and by extension their place or contributions in the community or health care system [21]. Understanding fundamental roles assumed by paramedics provides the profession, educators, as well as patients, the public and associated health or public safety professions/disciplines, with clarity and direction in how it may be most effectively realized, integrated and/or utilized.

Therefore, the objective of this study was to identify current and emerging roles all paramedics are to embody in Canada, regardless of designation or specialty, as defined by the profession. The aim was to be both reflective of current practice but also visionary in offering a conceptual framework that could promote discussion, debate and further study within the profession to ensure that what emerges ultimately has utility.

## Methods

### Study overview

This study involved a concurrent mixed methods design involving a focused discourse analysis (i.e., analysis of language used to describe paramedics and paramedicine) of peer reviewed and grey literature (Phase 1) [22] and in-depth one-on-one semi-structured interviews with key informants in Canadian paramedicine (Phase 2). Data from both methods were analyzed simultaneously throughout and after being merged using inductive thematic analysis [23–25]. Ethics approval for this study was obtained through the Justice Institute of British Columbia Research Ethics Board (JIBCER-201404) and informed consent to participate in this study was obtained by all participants.

For this research we adopted a constructionist approach [23, 25]. This research paradigm treats individual unique accounts and perspectives from authors/author groups and interviewees as valuable, rich and influenced by their specific but still meaningful context [26]. In applying this paradigm we accept that there are no hidden or “true” sets of roles to be discovered. Rather, any middle range theory or understanding that emerges regarding roles emerges as part of accepting that multiple views exist, which requires some degree of interpretation. As such our views and experiences are inextricably linked to the process. Knowledge and any roles that emerge are therefore co-constructed by the researcher-participant and researcher-literature interaction [27]. All authors are active researchers with extensive backgrounds in paramedicine as clinicians, educators, scientists, and/or regulators in Canadian paramedicine.

### Phase 1 data source: literature

Our intention was to explore the literature as sources of language used to describe paramedics and paramedicine. We specifically searched for language used by authors (while considering context) that might inform our understanding of roles directly, or through interpretation and themed analysis. In other words, we engaged in the literature as sources of language rather than as sources of evidence. Doing so involves an informed and iterative data driven exploration, rather than a highly specific or standardized search and/or evaluation of evidence. This discourse analysis then, is a qualitative analytic method that looks for “broad themes and functions of language” including “recurrent patterns or genres of language that share similar structures and content” [28] and/or a collective viewpoint. This use of *literature as data* focuses on the terms, phrases, descriptions and concepts within the documents rather than on the content and the arguments of the documents per se.

Two researchers (RB and BD) began by intentionally focusing on known seminal literature, both grey (defined as government reports, professional documents, and academic papers not controlled by commercial publishers) and peer reviewed, that has informed paramedicine in Canada broadly, including policy, operations, clinical practice, and education. Including both peer reviewed and grey literature emphasized our interest in language and recognized the presence, value and influential role of both in communicating, guiding and shaping the profession. Additional searches were conducted that used specific databases and search terms (see Table 1). We searched specifically for literature that (a) had at least in part, paramedics, paramedic practice or paramedicine as the focus of the work or unit or analysis; (b) discussed current or future paramedic practice; (c) described or made recommendations related to paramedics or paramedic practice; (d) discussed or informed the articulation of roles, functions, capabilities and/or attributes related to paramedics or paramedic practice.

**Table 1:** This table provides the search terms use as part of the discourse analysis

Category	Terms
Key Terms for Paramedicine	• EMS* • Emergency medical services* • Paramedic* • Ambulance • Prehospital • Pre-hospital
Secondary Terms	• Attributes • Capabilities • Competence* • Roles • Performance • Standards • Guidelines • Practice • Scope of practice • Themes • Trends • Change
Activity	# Articles
Initial Search (primary inclusion criteria)	817
Initial Full Article Review	25
Expanded Search (secondary inclusion criteria)	3,174
Title/Abstract Review	132
Final Articles for In-depth Analysis and Coding	94

* = a boolean search modifier that has the search engine return and highlight any word that begins with the root/stem of the word truncated by the asterisk

Initial data collection included a search of full-text English language literature published between1999 and 2014. While we focused initially on Canadian literature, we recognized the influence and increasing globalization of paramedicine, and included international works that had an influence on or application to Canadian contexts. We allowed the emerging data to generate framing and sensitizing concepts as they occurred, but remained open to any emerging ideas. We made efforts to be comprehensive and limit bias by seeking new and divergent ideas throughout. Both the literature and emerging concepts were used to iteratively seek additional data to strengthen, support, expand or challenge ideas.

### Data extraction and analysis

We searched for and extracted terms, phrases, extended quotes and concepts used by authors. The extraction process was informed by a series of related concepts. These included descriptions of paramedic practice, emerging trends, expectations of practitioners, identified gaps and recommendations, capabilities and attributes required of practitioners.

Coding was performed by two researchers (BD and RB), each of which read an overlapping subset of the full set of documents. The researchers employed an iterative process of data collection and open coding analysis, allowing for emergent themes to inform subsequent data collection and analysis [29]. A constant comparative method [23–25] was employed to further expand and refine codes. Memos, journaling and marginal notes were used to inform the coding structure but were also reviewed and integrated into the emerging data set. We continued this process until we reached saturation (i.e., no new codes or ideas emerged)) but stopped short of finalizing analyses until data from the literature could be merged and analyzed with interview data (discussed in more detail below).

### Phase 2 data source: interviews with stakeholders/Key informants

#### Overview

This phase of the study, which occurred concurrently with phase 1, involved in-depth one-on-one semi-structured interviews with key informants. Given the diversity of paramedic programs and models nationally, our sampling strategy was designed to ensure breadth in geography, specialties (tradition and non traditional) and stakeholders, while also using saturation of ideas as an end point. Interview questions broadly considered clinician level features and position in the health care/public safety fields that could be used to infer meaningful roles.

### Participants

We recruited and enrolled participants using purposive and snowball sampling strategies. We developed a nomination strategy to: (a) identify those recognized by the community to be most suited to speak on its behalf (e.g., valuable or unique insight, history of meaningful contributions, positions of influence); (b) ensure broad representation both in geography (e.g., provinces, mix of urban, rural, remote communities) and profession breadth (e.g., all levels of paramedicine, diversity in specialty units, service delivery models, stakeholder types).

We took advantage of a standing steering committee that was assembled by the PAC and the Alberta College of Paramedics to discuss revisions to the existing NOCPs. This committee included members from a number of stakeholder groups across Canada (e.g., regulators, educators, policy makers, employers, accreditors) and therefore was appropriately positioned as a starting point for our nomination and sampling strategies. We invited this group to offer nominations, but also to distribute the nomination request to other relevant respective provincial groups or individuals as they saw fit. We then rank ordered nominations against the criteria above to begin an initial set of invitations. Following each interview we asked participants whom we should additionally interview. We cross-referenced these ongoing nominations with our existing pool (and sampling goals), revising and adding participants as necessary.

### Interview guide

Interviews were one-on-one and semi-structured. The interview guide was developed (using consensus on initial questions), piloted and revised to ensure clarity and limit biases prior to beginning data collection. We made efforts to use open-ended non-judgmental questions and probes to inquire more deeply where appropriate. Our questions targeted (a) paramedics and the profession in the context of the health care and public safety sectors, (b) the position itself (across whatever context was relevant to the participant) and (c) the individuals who serve in the profession (regardless of specialization or position). We allowed the interview guide to remain open to revision throughout and in remaining flexible we allowed ourselves to discuss issues that had been raised in earlier interviews or on topics the participants wanted to explore. See Table 2 for the final interview guide.

**Table 2:** Interview guide for in depth one-on-one interviews with stakeholders and key informants

Health Care System	1. In what way do paramedics contribute to the health care system? 2. In what way should paramedics contribute to the health care system? 3. What role are they holding now? (e.g., in ER, Flu shots) 4. Where do you see similarities when considering paramedics and other health care professionals? 5. Where do you see differences when considering paramedics and other health care professionals? 6. What do you see as deficiencies in practicing paramedics in the health care system?
Position/Roles	7. At the entry to practice level, what would you like to see in paramedics? Or put differently, what should paramedics be able to do in order to fulfill some of the role(s) or functions we have been discussing? 8. What are “your” (e.g., stakeholder group, practitioner, public) expectations of a paramedic? a. As they interact with you/your organization? b. As independent professionals? c. As collaborative member of a paramedic team or multi-disciplinary team?
About the Individual	9. Think of the best paramedic or paramedics you know. What is it about these individuals that make them great? 10. Paramedics work is varied contexts and with a broad range of patients. What do you think is the difference between those that consistently function well and those that don’t? 11. What are common deficiencies or errors paramedics are guilty off that perhaps should be addressed? 12. What professional attributes do you see as essential for paramedics to maintain, develop or refine expertise over the duration of their careers? 13. What attributes or capabilities are integral in paramedic practice? (prompts: can you think of both clinical (i.e., those that are associated directly with patient interactions) and non-clinical (i.e., those that do are not directly associated with patient interactions) attributes?) 14. Do you have anything else you would like to share with us regarding attributes of paramedics in Canada from your point of view, regardless of level, specialization or position, now and to the year 2020?

### Data collection and management

Interviews were conducted primarily by WT with some conducted by RB and BD. Consent was obtained in writing and verbally in advance of the interview. All interviews were conducted over the telephone and audio recorded, and allowed to end naturally (i.e., no time limit). All recordings were then transcribed verbatim and reviewed for accuracy before analyses. QSR NVivo (http://www.qsrinternational.com/) was used to store, organize and analyze data.

### Data analysis and interpretation

All transcripts were coded by WT with a subset additionally coded by RB and BD. Open coding was conducted throughout to allow for the identification of areas requiring additional data and/or new lines of inquiry. This initial coding was intended to remain open to any possibilities that could be discerned in the data and to avoid any conceptual leaps as we moved through the analytical work. We used a constant comparative method to refine codes, and engaged in axial coding where we began to synthesize the data into more meaningful codes and groups until eventually themes or groupings (as well as sub groups or supporting codes) began to emerge. Similar to the literature review, we stopped short of completing the analysis, allowing for final analyses to be conducted once data had been merged with the codes/data from phase 1.

### Merging of the data and analysis

The initial analyses and coding in Phases 1 and 2 were merged into a single data set for further analysis. This was a deliberate attempt to allow both data sets, which had already been considered extensively (but not conclusively), and which we treated as equal, to be considered in relation to each other. This served as another and more extensive form of axial coding toward finalizing themes/roles.

The research team met repeatedly to carry out the analysis, sharing and merging of codes and data, looking for convergence, divergence and emergence of groupings or themes. This process was inductive, iterative and involved moving back and forth within and between the existing data sets (emerged codes and groupings, as well as original data) until both data sets were fully represented. The intent at this point was not to identify roles, per se, but rather to group together codes and data into conceptual or thematic categories. We avoided overly defining any themes that may have emerged, leaving raw or refined codes (and their associated raw data) that had been grouped to serve as our initial supporting data. We captured these analyses visually first using maps, then converted these into narrative text (derived or inferred from the data) summarizing these ideas as core concepts within each category.

## Results

### Part 1 - initial results from the literature

Using saturation as our end-point, we ultimately drew language from a total of 99 peer reviewed/academic (*n* = 77) and grey publications (*n* = 22). See Table 3 for a final list of publications. Five hundred and eight unique statements were extracted and coded. Preliminary axial coding and inductive thematic analyses (i.e., prior to merging of data with interview data) led to 49 initial emerging groupings before being merged with interview data.

**Table 3:** Literature review results

Peer Reviewed: 77				
Acker, Johnston, & Lazarsfeld-Jensen	2014	Industrial paramedics, out on site but not out of mind.	*Rural and Remote Health*	Australia
Armitage	2010	Role of paramedic mentors in an evolving profession.	*Journal of Paramedic Practice*	UK
Backe, Kaul, Klubmann, Liebers, Thim, Mabbeck, & Steinberg	2009	Assessment of salivary cortisol as stress marker in ambulance service personnel: Comparison between shifts working on mobile intensive care unit and patient transport ambulance.	*International Archives of Occupational and Environmental Health*	Germany
Ball	2004	Setting the scene for the paramedic in primary care: A review of the literature.	*Emergency Medicine Journal*	UK; English language literature
Brown, Dickison, Misselback, & Levine	2002	Longitudinal emergency medical technician attribute and demographic study (LEADS): An interim report.	*Prehospital Emergency Care*	USA
Brown, Margolis, Levine	2005	Peer evaluation of the professional behaviors of emergency medical technicians.	*Prehospital Disaster Medicine*	USA
Burges Watson, Sanoff, Mackintosh, Saver, Ford, Price,… Murtagh	2012	Evidence from the scene: Paramedic perspectives on involvement in out-of-hospital research.	*Annals of Emergency Medicine*	UK, USA
Campbell & Rasmussen	2012	Riding third: Social work in an ambulance.	*Health & Social Work*	Canada
Campeau	2008	Professionalism: Why paramedics require theories of practice.	*Journal of Emergency Primary Health Care*	None Stated
Campeau	2011	The paramedic kairotope theory: Findings.	*Journal of Paramedic* Practice	Canada
Clarke, Bradley, et al.	2014	Can paramedics use FRAX (the WHO Fracture Risk Assessment Tool) to help GPs improve future fracture risk in patients who fall? Protocol for a randomised controlled feasibility study.	British Medical Journal Open	UK
Commission on the Future of Health Care in Canada, & Romanow	2002	*Building on values: The future of health care in Canada: Final report.*	Commission on the Future of Health Care in Canada	Canada
Cooper	2005	Contemporary UK paramedical training and education. How do we train? How should we educate?	*Emergency Medicine Journal*	UK
Cooper & Grant	2009	New and emerging roles in out of hospital emergency care: A review of the international literature.	*International Emergency Nursing*	International literature
Cooper, Barrett, Black, Evans, Real, Williams & Wright	2004	The emerging role of the emergency care practitioner.	*Emergency Medicine Journal*	UK
Cummins, Garavan, Dixon, Landymore, Mulligan & O’Donnell	2013	The advanced paramedic clinical activity study (APCAS): An insight into the work of advanced paramedics in the mid-west of Ireland.	*Irish Journal of Medical Science*	Ireland
Dawson, King & Grantham	2013	Improving the hospital clinical handover between paramedics and emergency department staff in the deteriorating patient.	*Emergency Medicine Australia*	UK
Donaghy	2008	Higher education for paramedics – Why?	*Journal of Paramedic Practice*	UK
Donaghy	2008	Equipping the student for workplace changes in paramedic education.	*Journal of Paramedic Practice*	UK
Edwards	2011	Paramedic preceptor: Work readiness in graduate paramedics.	*Clinical Teacher*	Australia
Evans, McGovern, Birch & Newbury-Birch	2014	Which extended paramedic skills are making an impact in emergency care and can be related to the UK paramedics system? A systematic review of the literature.	*Emergency Medicine Journal*	English Language LIterature
Everden, Eardley, Lorgelly & Howe	2003	Emergency care: Change of pace.	*The Health Service Journal*	UK
Heardman	2014	Treating people with cardiac chest pain: Role of paramedics.	*Emergency Nurse: The Journal of the RCN Accident and Emergency Nursing Association*	UK
Hodge	2014	Developing leadership in the UK’s ambulance service: A review of the consultant paramedic role.	*Journal of Paramedic Practice*	UK
Hou, Rego & Service	2013	Review article: Paramedic education opportunities and challenges in Australia.	*Emergency Medicine Australia*	Australia
Hubble, Brown, Wilfong, Hertelendy, Benner & Richards	2010	A meta-analysis of prehospital airway control techniques part I: Orotracheal and nasotracheal intubation success rates.	*Prehospital Emergency Care*	English Language Literature
Jackson	2012	Senior paramedic role at North West ambulance service.	*Journal of Paramedic Practice*	?UK
Jensen, Croskerry & Travers	2011	Consensus on paramedic clinical decisions during high-acuity emergency calls: results of a Canadian Delphi study.	*Canadian Journal of Emergency Medicine*	Canada
Kilner	2004	Desirable attributes of ambulance technician, paramedic, and clinical supervisor: Findings from a Delphi study.	*Emergency Medicine Journal*	UK
Kilner	2004	Educating the ambulance technician, paramedic, and clinical supervisor: Using factor analysis to inform the curriculum.	*Emergency Medicine Journal*	UK
Lammers, Byrwa, Fales, Hale	2009	Simulation-based assessment of paramedic pediatric resuscitation skills.	*Prehospital Emergency Care*	USA
Landman, Lee, Sasson, Van Gelder & Curry	2012	Prehospital electronic patient care report systems: Early experiences from emergency medical services agency leaders.	*PloS one*	USA
Larkin & Fowler	2002	Essential ethics for EMS: Cardinal virtues and core principles.	*Emergency Medicine Clinics of North America*	Not declared; USA
Lazarsfeld-Jensen	2014	Telling stories out of school: Experiencing the paramedic’s oral traditions and role dissonance.	*Nurse Education in Practice*	Australia
Lord, Récoché, O’Connor, Yates & Service	2012	Paramedics’ perceptions of their role in palliative care: Analysis of focus group transcripts.	*Journal of Palliative Care*	Australia
Margolis, Romero, Fernandez, Studnek	2009	Strategies of high-performing paramedic educational programs.	*Prehospital Emergency Care*	USA
Mason, Wardrope, Perrin	2003	Developing a community paramedic practitioner intermediate care support scheme for older people with minor conditions.	*Emergency Medicine Journal*	UK
Mason, Coleman, O'Keeffe, Ratcliffe & Nicholl	2006	The evolution of the emergency care practitioner role in England: Experiences and impact.	*Emergency Medicine Journal*	UK
McClelland	2013	The research paramedic: A new role.	*Journal of Paramedic Practice*	UK
McKean	2013	The forensic paramedic: An outline of the role and essential criteria for the job.	*Journal of Paramedic Practice*	UK
Michau, Roberts, Williams & Boyle	2009	An investigation of theory-practice gap in undergraduate paramedic education.	*BioMedical Central Medical Education*	Australia
Mulholland, Barnett & Spencer	2014	Interprofessional learning and rural paramedic care.	*Rural & Remote Health*	Australia
Mulholland, O’Meara, Walker, Stirling & Tourle	2009	Multidisciplinary practice in action: The rural paramedic--it’s not only lights and sirens.	*Journal of Emergency Primary Health Care*	English language literature
O’Meara, Tourle, Stirling, Walker & Pedler	2012	Extending the paramedic role in rural Australia: A story of flexibility and innovation.	*Rural and Remote Health*	Australia
Pearson	2003	Emergency medical technician/paramedic’s role in prehospital drug delivery.	*Journal of Pharmacy Technology*	UK
Petter & Armitage	2012	Raising educational standards for the paramedic profession.	*Journal of Paramedic Practice*	UK
Reeve, Pashen, Mumme, De & Cheffins	2008	Expanding the role of paramedics in northern Queensland: An evaluation of population health training.	*The Australian Journal of Rural Health*	Australia
Roberts & Henderson	2009	Paramedic perceptions of their role, education, training and working relationships when attending cases of mental illness.	*Journal of Emergency Primary Health Care*	Australia
Ruple, Frazer, Hsieh, Bake & Freel	2005	The state of EMS education research project: Characteristics of EMS educators.	*Prehospital Emergency Care - Education and Practice*	USA
Safaei	2010	A ride to care – a non-emergency medical transportation service in rural British Columbia.	*Rural and Remote Health*	Canada
Shields & Flin	2013	Paramedics’ non-technical skills: A literature review.	*Emergency Medicine Journal*	UK; English language literature
Silversides	2009	Canada’s ability to respond to a national health crisis hampered by jurisdictional issues, untested emergency plans.	*Canadian Medical Association Journal*	Canada
Singh, MacDonald, Bronskill & Schull	2007	Interfacility transport of acutely-ill patients: Incidence of in-transit critical events.	*American Journal of Respiratory Critical Care Medicine*	USA
Singh, Ferguson, MacDonald, Stewart & Schull	2009	Ventilation practices and critical events during transport of ventilated patients outside of hospital: A retrospective cohort study.	*Prehospital Emergency Care*	Canada
Smart	2009	The role of the paramedic in health promotion.	*Journal of Paramedic Practice*	UK
Stevens &Alexander	2005	The impact of training and experience on EMS providers’ feelings towards pediatric emergencies in a rural state.	*Pediatric Emergency Care*	USA
Stevens, Jones, Smith, Nelson, Agho, Taylor & Raphael	2010	Determinants of paramedic response readiness for CBRNE threats.	*Biosecurity and Bioterrorism: Biodefense Strategy, Practice, and Science*	Australia
Studnek, Fernandez & Margolis	2009	Assessing continued cognitive competence among rural emergency medical technicians.	*Prehospital Emergency Care*	USA
Tam, Maloney, Gaboury, Verdon, Trickett, Ledu & Poirier	2009	Review of endotracheal intubations by Ottawa advanced care paramedics in Canada.	*Prehospital Emergency Care*	Canada
Tavares & Mausz	2013	Assessment of non-clinical attributes in paramedicine using multiple mini-interviews.	*Emergency Medicine Journal*	Canada
Tohira, Williams, Jacobs, Bremner & Finn	2013	The impact of new prehospital practitioners on ambulance transportation to the emergency department: A systematic review and meta-analysis.	*Emergency Medicine Journal*	UK; English language literature
Trede	2012	Becoming professional in the 21^st^ Century.	*Australasian Journal of Paramedicine*	None stated
Trojanowski & MacDonald	2011	Safe transport of patients with acute coronary syndrome or cardiogenic shock by skilled air medical crews.	*Prehospital Emergency Care*	Canada
Vilensky & MacDonald	2011	Communication errors in dispatch of air medical transport.	*Prehospital Emergency Care*	Canada
Vopelius-Feldt, Wood & Benger	2014	Critical care paramedics: Where is the evidence? A systematic review.	*Emergency Medicine Journal*	UK; International literature
Wang & Yealy	2006	Human patients or simulators for teaching endotracheal intubation: Whom are we fooling?	*Academic Emergency Medicine*	USA
Wang & Yealy	2006	How many attempts are required to accomplish out of hospital endotracheal intubation?	*Academic Emergency Medicine*	USA
Williams, Onsman & Brown	2010	Paramedic education: The significance of graduate attributes.	*Journal of Paramedic Practice*	Australia
Williams, Onsman & Brown	2012	A Rasch and factor analysis of a paramedic graduate attribute scale.	*Evaluation & the Health Professions*	Australia
Willis, Pointon, O’Meara, McCarthy & Lazarsfeld-Jensen	2009	Paramedic education: Developing depth through networks and evidence-based research – Executive summary.	*Australasian Journal of Paramedicine*	Australia
Woollard	2006	The role of the paramedic practitioner in the UK.	*Journal of Emergency Primary Health Care*	UK
Wyatt	2003	Paramedic practice – Knowledge invested in action.	*Journal of Emergency Primary Health Care*	Australia
Youngquist, Henderson, Gausche-Hill, Goodrich, Poore & Lewis	2008	Paramedic self-efficacy and skill retention in pediatric airway management.	*Academic Emergency Medicine*	USA
Grey Literature: 22				
Bigham, Brooks, Maher	2010	*Patient safety in emergency medical services: Advancing and aligning the culture of patient safety in EMS.*	Canadian Patient Safety Institute	Canada
Clarke, Harris, & Cowland	2010	Ethics and law for the paramedic.	Blaber, A. (2008). *Foundations for Paramedic Practice. A theoretical perspective*	UK
Commission on the Future of Health Care in Canada, & Romanow	2002	*Building on values: The future of health care in Canada: Final report.*	Commission on the Future of Health Care in Canada	Canada
Daly	2012	The paramedic in the community: My story.	*Primary Health Care*	UK
EMS Chiefs of Canada	2006	*The future of EMS in Canada: Defining the new road ahead*.	EMS Chiefs of Canada	Canada
Griffiths, Lowes & Henning	2010	*Pre-hospital anesthesia handbook.*		UK
Huot	2013	*Transition support for new graduate paramedics.*	Royal Roads University	Canada
Institute of Medicine	2007	*Emergency medical services at the crossroads*.	Institute of Medicine	USA
Jensen, Blanchard, Bigham, Dainty, Socha, Carter … & Morrison	2011	*Canadian National EMS Research Agenda.*	EMS Chiefs of Canada	Canada
Joint Royal Colleges Ambulance Liaison Committee	2000	*The future role of and education of paramedic ambulance service personnel.*	Joint Royal Colleges Ambulance Liaison Committee	UK
Lazarsfeld-Jensen, Bridges & Loftus	2011	Transitions: Command culture and autonomous paramedic practice.	*RIPPLE*	Australia
Margolis	2005	*The role of bachelor’s degree emergency medical services programs in the professionalization of paramedicine*.	Doctoral dissertation	USA
Morton-Cooper & Palmer	2000	*Mentoring, preceptorship and clinical supervision: A guide to professional support roles in clinical practice*.	Blackwell	UK
National Registry of Emergency Medical Technicians	2005	*2004 National EMS Practice Analysis*.	National Registry of Emergency Medical Technicians	USA
NHS Ambulance Chief Executive Group	2009	*Report of the National Steering Group on Clinical Leadership in the Ambulance Service*.	NHS Ambulance Chief Executive Group	UK
O'Meara, Walker, Stirling, Pedler, Tourle, Davis, Jennings, Mulholland & Wray	2006	O'Meara, P. Walker, J. Stirling, C. Pedler, D. Tourle, V. Davis, K. Jennings, P. Mulholland, P. Wray, D	Charles Sturt University	Australia
Page	2008	The quest for competence: What does it take to achieve competence in EMS?	*EMS Magazine*	USA
Page	2013	Clinical competence.	*Journal of Emergency Medical Services*	USA
Slade	2007	Occupational competencies for paramedic preceptors.	Master’s thesis	
Touchstone	2010	The importance of professional behavior.	*EMS World*	USA
Williams	2012	Keeping a ‘stiff upper lip’ in paramedic practice: Coping with emotion work.	*Nurse Education Today*	UK
Williams	2010	The individual, organizational, and system obstacles to patient-centric emergency medical services system design.	Master’s thesis	USA

### Part 2 results – interviews with stakeholder/Key informants

A total of 71 individuals were nominated from across Canada. After taking into consideration our sampling objectives and saturation, 20 stakeholders/key informants were interviewed in total, resulting in approximately 32 h of transcribed data. See Table 4 for a summary of participant demographics.

**Table 4:** Stakeholder and key informant demographics

Percentage of Females/Males	Females = 25 % (*n* = 5); Males 75 % (*n* = 15)
Average Years of Service	22 (SD = 8)
Provinces Represented	Nova Scotia (*n* = 2); New Brunswick (*n* = 1); Quebec (*n* = 3), Ontario (*n* = 7); Alberta (*n* = 2); British Columbia (*n* = 3), Federal (*n* = 2)
Services/Agency Types Represented (some participants hold multiple roles)	Ground (*n* = 10), Air (*n* = 4) and Military (*n* = 1) Paramedic Services; Educational Institutions (*n* = 8); Regulators (*n* = 1); Base Hospital (*n* = 3); Professional Associations (*n* = 5)
Operational Roles (some participants hold multiple roles)	Paramedic (*n* = 15); Paramedic (non-clinical) Administrator (e.g., chief, deputy chief) (*n* = 7); Paramedic Field Supervisor (*n* = 1); Educator (*n* = 9); Regulator (*n* = 3); CQI/Standards (*n* = 3), Paramedic Researcher (*n* = 4); Patient Advocate (*n* = 1); Medical Director (*n* = 3);
Practice Setting or Stakeholder Background (some participants hold multiple roles)	Land Paramedic Service (*n* = 12); Air Paramedic Service (*n* = 5); Rural (*n* = 2), Specialty (e.g., CBRNE) (*n* = 2); Military (*n* = 1), Community Paramedic (*n* = 2); International Paramedic (*n* = 3); Dispatch (*n* = 1); Emergency Department (*n* = 4); Other (*n* = 1)
Practitioner Group (some participants hold multiple roles)	PCP (*n* = 4); ACP (*n* = 11); CCP (*n* = 5); EMD (*n* = 1); Physician (*n* = 3); Nurse (*n* = 1), Other (*n* = 1)

*PCP* primary care paramedic, *ACP* advanced care paramedic, *CCP* critical care paramedic, *EMD* emergency medical dispatcher; Base Hospitals provide medical oversight and do not exist in all provinces

In total 321 codes were generated. As part of the axial coding process, open codes were then grouped based on conceptual similarity, resulting in a preliminary set of 16 groupings. Open codes and groupings were then merged with the results from the literature review.

### Merged results – framing concepts, roles and crosscutting themes

Our objective was to identify current and emerging roles paramedics are to embody in Canadian paramedicine. However, our analysis also revealed what we described as framing concepts (i.e., data that were not exactly roles but appeared to provide relevant context) roles and cross cutting themes (i.e., data that were similar to roles but appeared in all identified roles and thus were not distinct).

### Framing concepts

Three framing concepts emerged: (a) variable context of practice, (b) embedded relationships and (c) a health and social continuum.

#### Variable context of practice

Paramedicine was discussed in ways that suggest shifts in the profession. These shifts occurred in terms of practice location (e.g., paramedics working in emergency department or clinics) [30] but also models of care [1, 11, 31]. For instance, paramedicine was no longer viewed as exclusively emergency response based, offering more in terms of contributions to health and health care:

*“I believe that the paramedic profession is at a point of maturation that it is well beyond the singular focus of the resuscitation days…we actually have a greater role to play, with more significant dividends in the areas of prevention, education and primary care.”[P-13]* Emergency care was certainly an emphasis, but now for different reasons: *“our primary focus is an emergency, likely probably will remain an emergency, but the more work we do on the primary healthcare side, the less I suspect there will be on the emergency side.”[P-09]* This is leading to variable work and models of care that increasingly represent deviations from or expansions on traditional emergency care and opportunities: *“Paramedicine will increasingly develop a skill set that would permit a larger range of problems to be managed without hospital visits.”[P-AL]* Even when engaged in emergency response this shift included empowering paramedics to provide more patient centered dispositions. Shifts in physical workspace and models of care, novel contributions to health care and more patient centered care collectively served as one framing concept that helped to provide context for some of the roles described below.

#### Embedded relationships

A second framing concept involved paramedic clinical practice as being embedded in a number of relationships. Examples included relationships with patients, their family, their health history and existing care plans (if any) [30], with other healthcare team members (directly or indirectly) [1, 3, 30–32], their (i.e., both patients and paramedics) position in the larger healthcare system as well as social and/or cultural context [33]: “*paramedics are part of a systems approach to health care, so a community system of care, an out of hospital system of care, and a hospital system of care, and they overlap.”[P-02]* Others have discussed how inter-dependencies between the health care services have created a need for paramedicine to foster strong linkages with other stakeholders, including other health care professionals and regions [1], even suggesting that performance is dependent on being able to function in such a system: *“[paramedics] are a part of a network of services…connected to a continuum of care. And so I think the ones that do well in the varied contexts are the ones that see themselves as part of a network.”[P-01]* These relationships were positioned as ways of optimizing care and promoting patient centeredness while taking advantage of the unique point of contact: *“you elevate the relationship of the paramedic and whichever specialist they are collaborating with in an interprofessional model to enhance that patient’s experience.” [P-13]* The central thesis being that healthcare begins or continues with paramedics rather than serving as a bridge to healthcare and that paramedics have the added advantage of engaging with patients in their particular context. However, again, it appears these contexts are not to be considered in isolation, rather as embedded in healthcare delivery [1, 30].

#### Health and social continuum

Finally, participants and the literature describe paramedic-patient encounters as involving mutually dependent health and social factors, although differing in the degree and relevance across instances [33]. This was discussed in terms of ensuring care considers both medical and social (and psychological) determinants and that paramedics be equipped to consider these: *“whether it is chest pain or respiratory distress. It can also be a social issue…the paramedic has to be very comfortable with that skill set as well. So with respect to not only the usual, you know, diagnosis and management, but the social aspects of health care…therefore you have to further develop your skill sets with non-urgent things, like managing blood pressure, CHF care, maybe even ongoing social issues.”[P-07]* A precipitating health problem may have initiated contact with paramedics, but in some cases this may be secondary to underlying social/psychosocial determinants of health. This was also revealed when discussing an apparent dissonance between how paramedics are trained (referring to the exclusions of social factors) and what is involved or required in their daily activities with patients in their context in order to better serve their needs [33]: *“[The] system and the curriculum and the NOCP’s, we systematically herald one arm - which is the 911 [emergencies]- and at the same time, discount the process where the majority of the calls are going to be.”[P-19]* It appears therefore that there an a growing appreciation for (or call for more attention to) being prepared for, or acting on the range patient needs beyond acute events and recognising any events in the individuals social context.

### Roles paramedics are to embody

These framing concepts help to provide context and in some ways even explain six distinct roles to be embodied by paramedics. These were, (a) clinician, (b) team member, (c) health and social advocate, (d) educator (e) reflective practitioner and (f) professional.

#### Clinician

Perhaps most consistent with existing models of paramedicine was that paramedics need to be able to provide safe and effective clinical care using knowledge, skills and clinical judgment within a given scope and standards of practice, while drawing on and functioning within the unique contexts in which they practice [1, 2, 34]. However this applied to emergency and non-emergency patient care needs, patient disposition and a broader range of patient types or interventions: *“they [paramedics] must be able to deal with the undifferentiated early in their care…and triage them to the most appropriate resource available.”[P-07] “I think that paramedicine will increasingly develop a skill set that would permit a larger range of problems to be managed without hospital visits.”[P-01] “They must be able to make a field diagnosis…and actually make a treatment decision in terms of where the patient should go. Is there something we can treat them right here as opposed to you know bringing them to the hospital.”[P-11]* These concepts of a clinical proficiency were reflective of current practice models but also of practice models that were evolving. Technical competence [3, 35], being patient-centered [36], having the ability to integrate a patient’s context and functioning in diverse settings with varying levels of clinical support [37] were also features of the discourse in this “Clinician” role. However, given the diverse patient groups and contexts, it wasn’t clear whether specialists or generalists would be most appropriate or what defines competence exactly, only that it was an essential role to be embodied in this system.

#### Team member

Both the literature and participants described the integration of care provided by paramedics as part of a patient’s overall healthcare as an emerging and growing shift in the profession. This concept extends the clinician role by emphasizing safe and effective *integrated* healthcare, where paramedics are functionally involved in and responsible for health and health care systems (or networks) of care and providing team-based care in broad interprofessional settings [1, 32]: *“This expansion of their scope and role is predicated on a collaborative practice model that they can't do in isolation, they need to have this integrated teamwork.” [P-02]* Engaging paramedics into these integrated and collaborative heath networks was discussed at the system level, but with implications for paramedicine. Optimizing care meant, for some, recognizing that paramedics work (or should work) within networks of services, within a community connected to a continuum of care: *“I think with paramedicine there’s going to be a lot more interaction with doctors and nurses and less transport for the benefit of patients. And I think that’s going to be a very huge component…I think we’re going to have to look at all sorts of alternates for the system to function properly.” [P-16] “This involves inter-professional collaboration and how to work appropriately with nursing and dietary and medicine and physiotherapy…and all those other health care professions.” [P-15]* This “Team Member” role included language related to a deeper understanding of the broader healthcare system and the ability to work in multiple forms of collaborative environments, building short and long-term relationships with patients and family members, as well as other responders and health providers to meet common goals [3, 32]. Abilities related to shared leadership, collaboration, and synchronous and asynchronous teamwork were also described in this context of being an effective team member [31, 32].

#### Health and social advocate

Paramedics were discussed as having a privileged and unique point of contact, offering the opportunity to address a patient’s medical needs while taking into consideration their unique social context [33, 38]. This suggested opportunities for navigating a patient’s care or disposition collaboratively in an advocate-like way that considered both health and social needs or context: *“That’s a privileged position which I think makes paramedics more unique from most health care professionals in the system is that they are privileged to be the first line contact with the patient and within the community.”[P-19] “I think that the future would be being able to empower the paramedics to be able to determine where that person needs to go. Can they stay put in the client’s home safely? Do they need to go the community health centre? Do they need to go directly to the x-ray for diagnostic testing? Or should they go the ED (emergency department).”[P-12]* This Health and Social Advocate role, albeit a somewhat untraditional form of advocacy, emphasized social context in the health and social continuum (described above) and to some extent obligates paramedics to act, if equipped with the knowledge to do so. This appeared to be in response to system level challenges of the day, that is the challenges some patient’s might face in accessing or optimizing their care: *“We have become a bit of a safety net of the overall healthcare system in that when they can’t find a physician, they turn to paramedics…or becoming the eyes and ears of the of the primary [healthcare] system as well. I think there’s a role for - for paramedics to start to close some of those gaps that are out there, so that hopefully in the long term it will result in better primary care.” [P-14]* Paramedics must therefore be able to detect and incorporate social features (e.g., physical environment, cultural context) and health status (e.g., ongoing health history, existing care plans) to employ shared decision-making and collaborative leadership in serving both as health champions and social advocates.

#### Educator

This role emerged in two ways. First, paramedic-patient interactions were viewed as unique opportunities to promote patient health by providing timely health teaching when interacting with patients in the community [1, 30, 39]. This was again linked to taking advantage of opportunities associated with interacting with patients in their own contexts: *“from the preventative side…trying to mitigate the impact on the health care system overall by being proactive in health education and health promotion.” [P-12] “You have this wonderful ability to go and screen and identify these environmental or contextual or health relationships, or health environments that the patient is in. During the care, they have this marvellous opportunity of education-the teachable moment.”[P-02]* Second, this “Educator” role was also discussed in terms of being able to seek teaching and learning opportunities in daily practice settings and in every interaction with patients, but also for colleagues (including trainees), other responders and health or public safety providers. Some argued that abilities such as integrating experience and evidence to develop and share tacit (professional) knowledge are essential and contribute to ongoing development of paramedic practice and understanding [40]: *“I look for those that share that knowledge and who take on that teaching role and will commit to, when they see something that maybe could be done differently among their peers…go to the them afterward and have that teachable moment with them in a way that that person walks away better for it and shares that knowledge with them.[P-14].* Being able to serve larger goals of health promotion and disease prevention (or exacerbations), as well as advancement of the profession on a daily basis were seen as opportunities and requirements in practice.

#### Reflective practitioner

Paramedicine is an autonomous, evolving and ever changing profession with expanding expectations in complex and rich practice contexts [1, 3, 32, 36, 41]. Linked to some extent with other roles (e.g., clinician) many discussed an obligation to be reflective about one’s practice. This was described as the ability to integrate experience, evolve personally and professionally through self-awareness, self-monitoring, and self-reflection (reflecting in and on action) as way of optimizing patient care and their own physical, mental and emotional well being [3, 40]. *“Engage in the self-refection and the reflective practice pieces: Did I connect with the patient? What was good? What did I maybe miss? How could I have been a better advocate? How was my interaction with the rest of the health care team? How can I follow up? Who can I connect that patient to in the community or at the hospital? [P-19] “…and that they have competence with resilience, or some understanding of self-regulation, self-care, and that that matters as well”.[P-19]* This “Reflective Practitioner” role was discussed as the ability to function within their scope, while also being accountable to and informed by evidence and best practices and taking corrective action when necessary. This role was reflected in statements related to having a culture of curiosity and engaging in (and being equipped for) life-long learning, integrating the individual’s ongoing experience and evolving practice to develop and maintain professional and clinical acumen. “*The culture of curiosity-I call it-is when you read up on things; you dig deeper into things; you go to the literature… to start asking, but also to say ‘I want to know more about this…that whole desire to continue to learn throughout the course of your life. As opposed to being ‘fed.”[P-15]* In the context of profession growth, data suggested a greater emphasis be placed on engaging in learning (and research) within the moment and across their careers to address gaps, maintain currency, and extend their expertise. This was recognized as dependent on access and opportunities to learn within their communities of practice, including from their peers, employers, regulatory bodies, and profession. This role, unlike the others was closely tied to and difficult to separate at times from our final role, discussed next.

#### Professional

The expectation that paramedics embody various attributes traditionally associated with being professional also emerged. Often this was positioned in the context of the profession’s unique position in the community, the autonomy of work they engage in, the development of the profession and in establishing or maintaining trust from the community/society. A number of core attributes were discussed including behaving ethically, morally, with integrity and respecting the individual while avoiding further harm: *“Follow a code of professional conduct…that speaks to practicing competently, to you know being a mentor and teaching and being taught…keeping the patient as your primary focus and concern and recognizing your limitations” [P-14] “These are the things that matter: compassion, empathy, professionalism.”[P-19] “Professionals appreciate and understand what it means to be an ethical and moral health provider.”[P-14]* These and other attributes (e.g., honesty, care, compassion, empathy, continued commitment to excellence) [3, 42, 43] were closely linked to informing the practitioner’s performance, patient and professional interactions, and their relationship to the profession and society overall [3]. Finally, there were also undertones signaling a required shift such as engaging in more research, being more responsible for one’s own ongoing development and habitual maintenance of competence, as a way of supporting this role. Research for example was associated with being a professional within a profession: “*People are not graduating in EMS with an appreciation or understanding of research and one of the best ways to move that health system forward, they should be able to contribute back in the form of knowledge and if we could start to do more of that, it would be fantastic. And start to generate and build our own body of research*”. [P-14] Characteristics, behaviors and activities all informed this role as ways of being, promoting ways of practicing optimally and promoting development and growth of the profession.

### Crosscutting themes

While over-arching framing concepts provided context and were informative in appreciating how the roles emerged and were discussed, four other themes emerged in the data that again were not quite roles but almost always present in what roles did emerge, and therefore were “crosscutting” in type. These were “patient safety”, “compassion”, “adaptability” and “communication”. In the stakeholder interviews in particular but also across a number of publications, [1, 3, 31, 32, 34, 37, 40, 43–46] these concepts were discussed but with varying emphasis across the roles. For instance within the clinician role and team members roles, patient safety was discussed as something to be expected, unquestionably, and that quality care and promoting compassion and communication be the developmental focus. These themes emerged not just in the roles, but in the framing concepts as well. Adaptability for example emerged in discussion regarding shifts in practice and embedded relationships. If these roles are to be operationalized, it will be important to consider how each is supported by or includes each of the crosscutting concepts.

### Conceptual model

**Fig. 1:**
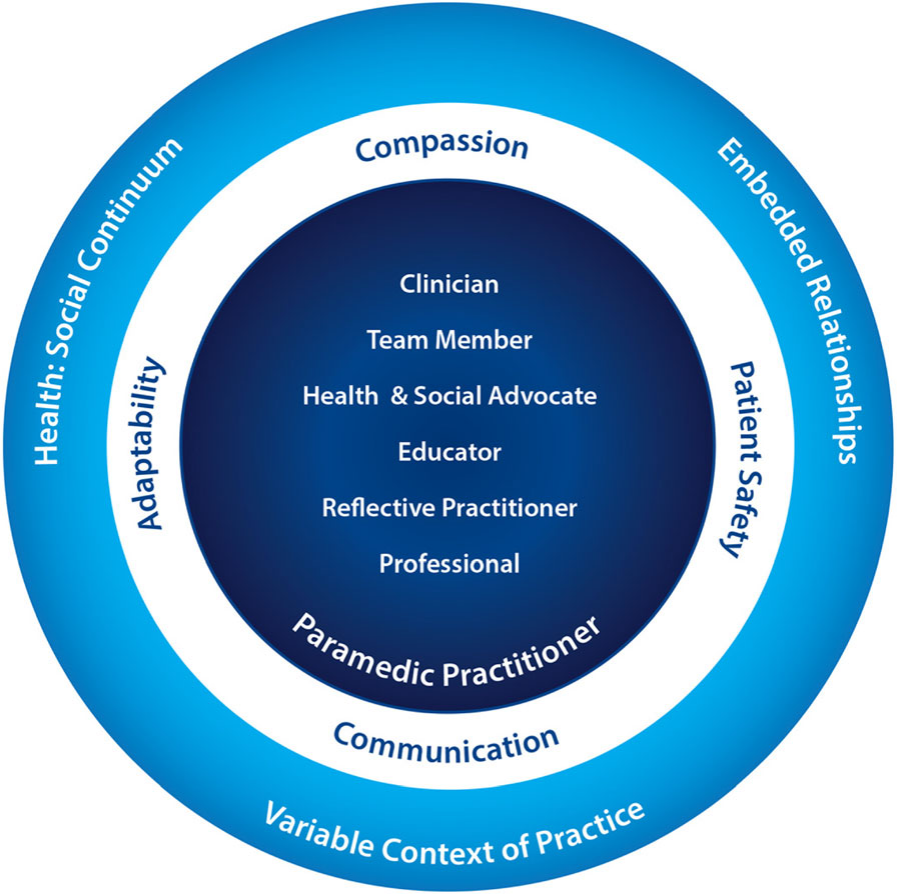
Graphic representation of conceptual model that emerged from our data; moving from the outer circle in are framing concepts, crosscutting themes and roles paramedics at all levels or specialization are to embody

Our results are represented visually in Fig. 1 illustrate our conceptual model and the relationship between findings. The center represents each of the distinct and interacting roles (as described above) that together constitute the paramedic. Working outward, the next ring highlights the crosscutting themes in which all roles are to function within. The most outer ring provides the context for the inner ring and centre, to describe the setting which all of these findings appear to exist.

## Discussion

This study aimed to identify and ultimately communicate potential roles to be embodied by paramedics (in Canada), regardless of designation or specialty. Following a discourse analysis of peer and grey literature and in-depth interviews with stakeholders and key informants, six “roles” emerged that may be reflective of present day practice and/or what the profession considers meaningful to practice moving forward. These include, “clinician”, “team member”, “health and social advocate”, “educator”, reflective practitioner” and “professional”. Framing concepts, which provide some context for these roles, also emerged, including “variable contexts of practice”, “embedded relationships” and the concept of a “health and social continuum”. Lastly, cross cutting themes including “patient safety”, “adaptability”, “compassion” and “communication” appear to exist in all roles. This study contributes a novel synthesis and elaboration of paramedic practice and collectively, these roles, framing concepts and cross cutting themes provide a preliminary and foundational conceptual model for how paramedics (and paramedicine) may be defined and/or described in Canada.

The findings presented here share similarities with a number of other health profession frameworks, particularly at the roles level. For example, the Royal College of Physicians and Surgeons of Canada CanMEDs framework, [47] the College of Nurses of Ontario, [48] the National Physiotherapy Advisory Group, [49] Canadian National Dental Hygienist Group, [50] and the Profile for Occupational Therapy Practice in Canada, [51] all share a number of roles to be embodied by their respective clinicians. The consistency across health disciplines, including now paramedicine, suggests common foundational concepts required of health professionals (e.g., communication, safety, clinical competence). However, whether roles are defined differently, yet use the same name, or unique roles are identified, being responsive to and reflective of the associated profession is necessary to ensure relevant, meaningful and unique contributions to the health care system. Our inductive strategy, consistency with other health professions and yet unique and context specific results, may provide a degree of support for our findings.

Similar to other health professions, [47] while each role was distinct we did observe a degree of intersecting. That is, each of the roles was dependent on or included aspects of others to varying degrees. For example, the clinician role was associated with and dependent on being a reflective practitioner, which was associated with being a professional and so on. These intersections suggested that paramedics must be able to perform each role fully but also integrate and adapt all roles in practice in order to meet patients’ health and social needs in uncontrolled, diverse and unpredictable contexts of practice. This has implications for practice, but also educators and the assessment of competence. While distinct, none of the roles were so distinct as to function in complete isolation of the others and optimizing practice (and patient centered care) is likely to involve varying degrees of each across interactions. The degree to which this intersecting or overlap occurred varied by roles and their combinations, but was almost always present and therefore any emerging model (or operationalization of a model) must take into consideration these findings in order to have utility.

Even when recognizing the dependence on or relationship between roles, one of the common themes in our findings was that this redefined “paramedic practitioner” (i.e., one who embodies all roles) emerged with undertones of a profession struggling with a dissonance between how it is currently realized and its actual or optimal place in the health care system. While staying true to the origins of the profession and the need or desire to continue to serve the emergent and urgent care needs of the public, there was an emphasis placed on the profession’s unique context of practice and potential (perhaps yet to be fully realized) role in healthcare. For example, the “team member” and “health and social” advocate roles were reflective of this tension. We learned that for many, healthcare for patients in the community specifically, might be enhanced or optimized by integrating paramedics (i.e., team member role) and/or by having paramedics serve as navigators of care (where appropriate) (i.e., health and social advocate). These shifts are already underway [8, 52, 53]. The diversity of care and the relevance and involvement of social influences, has it appears, become more and more dominant in the interactions paramedics are having with patients. As a result, much of what eventually formed the roles that emerged was informed or at least influenced by this tension. However, whether the adoption or implementation of these concepts result in improved patient outcomes, filling existing gaps, improved health care services or efficiencies, safer or better care requires further study.

In light of this tension, the roles identified here were communicated by the profession (not the public, other health professions, nor patient groups) and were in a way socially constructed. This carries with it at least three submissions to consider. Other health professions that have worked through similar roles frameworks, especially early in their implementation, have documented challenges unique to their settings [54, 55]. First, while perhaps conceptually appealing, for some regions (e.g., urban vs. rural, where resource limitations exist) or existing specializations (e.g., flight, marine, heavy urban search and rescue) some of these roles may be limited in utility and/or difficult to operationalize. Whether alternative dispositions for example (a significant feature of what health and social advocate includes) or truly integrating as members of a larger healthcare team can be achieved or have relevance, may be a limiting factor. Further, socially constructed frameworks may intentionally or unintentionally omit or include certain aspects of practice. However, specializations like the ones described above are built on (or deviate from) a foundational and entry level set of roles and therefore does not inherently diminish their relevance, but may become less relevant as specialty care/service become the focus. How these findings ultimately compliment, augment, support or deviate from other health professions and/or public expectations requires consideration.

Second, before these roles become more formally part of or contribute to any profession-defining or guiding framework, they must be further studied and validated using additional independent and rigorous research. That is, researchers, policy makers, educators and practitioners must critically analyze and explore whether these roles can be further quantitatively or qualitatively supported (or modified) using other existing data or prospectively. Legislative, educational, professional and other potential challenges will also need to be explored for alignment and feasibility.

Third, these roles will require supporting documents in order to be fully realized. For instance, this may include standards of practice to inform boundaries (e.g., for the “clinician” role), a curriculum framework to support educators/educational institutions in operationalizing the ideas, an assessment framework to support employers and regulators in ensuring candidates have indeed achieved or have the capabilities, entrustable professional activities or milestones necessary to embody each interacting role to an established standard.. These issues do not immediately invalidate these results; rather, it begins to suggest where areas of development may be needed in order to place these roles into practice.

The model presented here and the surrounding concepts signal a place to begin more targeted discussions (and research) about the existing and/or intended roles, crosscutting themes and framing concepts associated with paramedics and paramedicine in Canada. This may then begin to more clearly articulate the paramedic practitioner’s place in the larger healthcare system and how this once mainly emergency care based profession is now far more complex and multi-faceted.

### Limitations

This study is associated with some limitations. This study explored a range of concepts and expectations that describe current and emerging paramedic practice by selected stakeholders and literature. Our initial searches found relatively little peer-reviewed literature that spoke directly to the functions and roles expected of paramedics in Canada. As such we intentionally focused on the discourse and language used by authors who come from a variety of backgrounds, with varying goals and when writing from differing perspectives. Second, while we made efforts to ensure a majority of stakeholders were represented and that those who contributed on behalf of those stakeholder groups were appropriately selected (as per our nomination strategy), some may argue that selected stakeholder groups or specializations (or other demographics) may not have been adequately represented. For example, we may have under-represented primary care paramedics or those working in rural-remote areas, and over-represented leaders in the profession or allied health professions that are closely related to paramedicine (e.g., nurses, emergency physicians). We acknowledge this possibility and have therefore presented our results not as final but as a starting point for discussion, further exploration, ongoing study and validation.

## Conclusion

The paramedic profession in Canada is experiencing a shift that appears to be grounded in an appreciation for the unique context of practice, embedded contexts and relationships and health and social continuum that paramedics assume in their daily practice settings. Paramedics may need to demonstrate capabilities supporting six roles (clinician, team member, leader at the patient’s side/health and social advocate, educator, reflective practitioner, professional) in order to optimally meet the needs of patients they encounter in the community and support healthcare system goals. Whether or how these roles have utility and/or can be fully realized or operationalized will require further study. However, the three framing concepts, six roles and four cross cutting themes provide a foundation by which to engage in such study, reflects the potential for a more complex and multifaceted health discipline and may contribute toward a national profession defining or guiding framework.

## Abbreviations

ACP: Advanced care paramedic
BD: (Author) Becky Donelon
NOCP: National occupational competency profile
P: Participant
PAC: Paramedic Association of Canada
PCP: Primary care paramedic
RB: (Author) Ron Bowles
WT: (Author) Walter Tavares
